# S6AE: Securing 6LoWPAN Using Authenticated Encryption Scheme

**DOI:** 10.3390/s20092707

**Published:** 2020-05-09

**Authors:** Muhammad Tanveer, Ghulam Abbas, Ziaul Haq Abbas, Muhammad Waqas, Fazal Muhammad, Sunghwan Kim

**Affiliations:** 1 Telecommunications and Networking (TeleCoN) Research Lab, GIK Institute of Engineering Sciences and Technology, Topi 23640, Pakistan; tanveer.m@giki.edu.pk; 2 Faculty of Computer Science and Engineering, GIK Institute of Engineering Sciences and Technology, Topi 23640, Pakistan; abbasg@giki.edu.pk (G.A.); engr.waqas2079@gmail.com (M.W.); 3 Faculty of Electrical Engineering, GIK Institute of Engineering Sciences and Technology, Topi 23640, Pakistan; ziaul.h.abbas@giki.edu.pk; 4 Faculty of Information Technology, Beijing University of Technology, Beijing 100124, China; 5 Department of Electrical Engineering, City University of Science and Information Technology, Peshawar 25000, Pakistan; fazal.muhammad@cusit.edu.pk; 6 School of Electrical Engineering, University of Ulsan, Ulsan 44610, Korea

**Keywords:** IPv6 over Low Power Wireless Personal Area Networks, security, authentication and key exchange

## Abstract

IPv6 over Low Power Wireless Personal Area Networks (6LoWPAN) has an ample share in the Internet of Things. Sensor nodes in 6LoWPAN collect vital information from the environment and transmit to a central server through the public Internet. Therefore, it is inevitable to secure communications and allow legitimate sensor nodes to access network resources. This paper presents a lightweight Authentication and Key Exchange (AKE) scheme for 6LoWPAN using an authenticated encryption algorithm and hash function. Upon successful authentication, sensor nodes and the central server can establish the secret key for secure communications. The proposed scheme ensures header verification during the AKE process without using IP security protocol and, thus, has low communication and computational overheads. The logical correctness of the proposed scheme is validated through Burrows–Abadi–Needham logic. Furthermore, automatic security analyses by using AVISPA illustrate that the proposed scheme is resistant to various malicious attacks in 6LoWPANs.

## 1. Introduction

Low Power Wireless Personal Area Networks (LoWPANs) are an essential part of the Internet of Things (IoT) and are composed of resource-constrained devices tractable with the IEEE 802.15.4 standard. LoWPAN is a promising technology [1,2] having potential applications in smart grids, home automation, e-health-care, battlefield, and security surveillance. Such networks are constricted in storage capacity, transmission range, computational capabilities, power resources, and data rate. To provide Internet connectivity to LoWPAN devices, IPv6 is considered to be the most accordant solution [3,4]. However, IPv6 is a resource-intensive protocol originally designed for desktop and server environments and has a maximum frame size of 1280 bytes, whereas the maximum physical layer frame size for IEEE 802.15.4 is 127 bytes [5,6].

To make IPv6 frame size tractable with the IEEE 802.15.4 physical layer, the Internet engineering task force has standardized an IPv6 over LoWPAN (6LoWPAN) adaption layer [7]. This layer provides IPv6 packet fragmentation, encapsulation, reassembly, and header compression mechanisms [5,8]. In addition, 6LoWPAN renders the functionality for seamless transmission of IPv6 packets across networks and provides a mechanism for stateless addressing.

Sensor nodes deployed in a 6LoWPAN network are used to accumulate vital information from surrounding environments and transmit the collected information to a central location. Thus, for a streamlined operation of 6LoWPANs, confidentiality and integrity of the transmitted information must be ensured [9,10]. However, the original 6LoWPAN design does not include security and privacy features. The following subsection reviews eminent proposals for securing 6LoWPANs.

### 1.1. Related Work

Cryptographic encryption techniques and message validation mechanisms are applied for securing communications in 6LoWPANs. For this purpose, an Authentication and Key Exchange (AKE) scheme is essential before applying the cryptographic algorithms. An AKE scheme ensures the legitimacy of sensor nodes deployed in 6LoWPANs and also establishes a secret session key to protect communications between sensor nodes and the server from an attacker [11].

The authors in [12] propose a lightweight IP Security (IPsec) based scheme for 6LoWPANs to achieve secure end-to-end communication. The scheme introduces a pre-shared key concept for AKE, but it does not provide any information about the session initialization and secure mobility. In [13], the authors present a scheme, called scalable security with symmetric keys, to achieve secure communication among end-devices in IoT. However, the scheme is not feasible for large 6LoWPANs because of its higher computational overhead and complex key management process. The authors in [14] propose a Secure Password Authentication Mechanism (SPAM), which supports a secure handover process in the proxy mobile IPv6 networks. However, the main drawback of the SPAM mechanism is the higher transmission delay incurred as a result of the re-authentication process. In [15], the authors propose a mechanism that renders security for wireless sensor networks by deriving the key from a pre-distributed master key. However, the scheme is computationally expensive for resource-constrained devices. The authors in [16] propose a Secure Authentication and Key Establishment Scheme (SAKES), which is based on public-key cryptography, but it is computationally expensive for resource constricted devices. Additionally, SAKES does not provide secure mobility and is vulnerable to the node compromised attack. To reduce the computational burden on sensor nodes, SAKES performs most of the computation at gateway nodes and sends the computed key to sensor nodes in the 6LoWPAN environment. Therefore, to perform the handover, it is necessary for sensor nodes to start the new key establishment process. Furthermore, SAKES assumes all sensor nodes in the 6LoWPAN environment to be static. The authors in [17] propose an Efficient Authentication Key Exchange for 6LoWPAN (EAKES6Lo) based on the Elliptic Curve Cryptography (ECC) and Advance Encryption Standard Counter Mode (AES-CTR-128). EAKES6Lo ensures secure session key establishment and provides mutual authentication between sensor nodes and the server. However, EAKES6Lo is computationally expensive and does not provide privacy. In [18], the authors propose a Lightweight Authentication Protocol (LAUP), which is based on the symmetric key cryptosystem while using the pre-shared key mechanism. LAUP is time efficient and consumes low power during the AKE process. However, the scheme does not provide effective measures for secure mobility.

The authors in [19] propose a communication security and privacy support based on the public key cryptography and pre-shared key mechanism to achieve secure communication in 6LoWPANs. The authors in [20] propose a 6LowPSec security protocol that provides end-to-end security among 6LoWPAN nodes using the existing hardware security mechanism specified by IEEE 802.15.4 Media Access Control (MAC) sub-layer. However, their proposed security protocol does not provide mobility support and header verification.

### 1.2. Contribution and Paper Organization

This paper proposes a lightweight AKE scheme, called Securing 6LoWPAN using Authenticated Encryption Scheme (S6AE), which provides mutual authentication between the server and sensor nodes and also ensures header verification during the authentication process without employing the IPSec protocol. S6AE employs the well-known ASCON algorithm for authenticated encryption in 6LoWPANs. To the best of our knowledge, ASCON has never been employed in the literature for securing 6LoWPANs. Additionally, S6AE employs SHA-256 hash function and bit-wise XOR operations to achieve AKE in 6LoWPANs. SHA-256 is used to generate unique output strings by using the S6AE secret parameters. To decrease the communication overhead by means of reducing the message size, the length of the SHA-256 output string must be reduced to 64-bits with minimum computational cost and without compromising performance. For this purpose, we use bit-wise XOR operations. The key contributions of this paper are listed below.

- The proposed scheme provides end-to-end security, mobility support, and header integrity.
- Informal security analysis and formal validations using Burrows–Abadi–Needham (BAN) logic and Automated Validation of Internet Security Protocols and Applications (AVISPA) illustrate that S6AE secures 6LoWPANs against various malicious attacks.
- Comparative analysis with eminent existing schemes demonstrates that S6AE is more efficient and provides better security features with less computational and communication overheads, memory utilization, and energy consumption.

The remainder of the paper is organized as follows. System models and preliminaries are discussed in Section 2. Section 3 details the proposed S6AE scheme, and Section 4 provides security analysis of S6AE scheme. Performance evaluation is presented in Section 5. Finally, the paper is concluded in Section 6.

## 2. System Models

This section presents the models and preliminaries used in the proposed scheme.

### 2.1. Network Model and Security Assumptions

**Figure 1:**
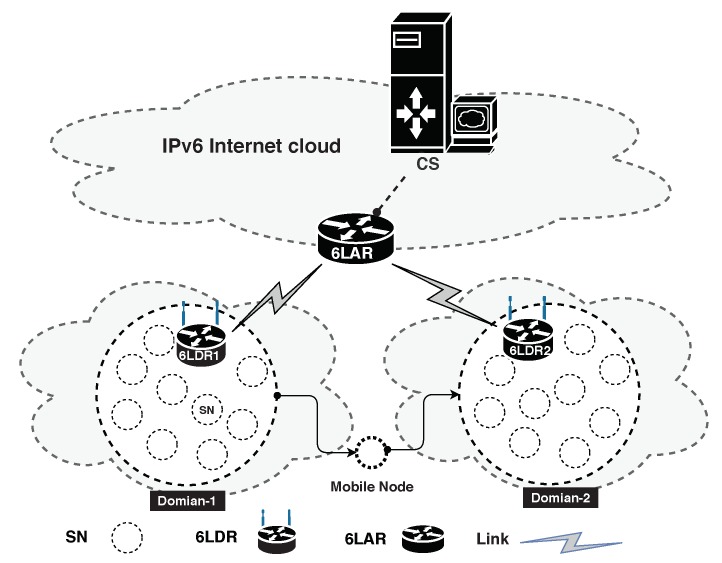
6LoWPAN network architecture.

This paper considers the network model shown in Figure 1 for the authentication process. The 6LoWPAN network model consists of sensor nodes (SNs), domain router 6LDR, the access router (6LAR), and the central server (CS). SNs are used to accumulate information from the surrounding environment and transfer the collected data to CS for further processing. Moreover, 6LDR provides Internet connectivity by SNs in a domain. 6LAR provides inter-connectivity with CS in IPv6 cloud. It is assumed that the communications among 6LAR, 6LDR, and CS are secure. Besides, it is assumed that CS is reachable by SNs, 6LDR, and 6LAR. 6LDR registers itself with CS through a secure channel. Additionally, SNs and 6LDR exchange their pseudo-identities (SIDs) through neighbor discovery (ND) protocol. Furthermore, each 6LDR registers itself with 6LAR. All the devices in 6LoWPAN learn about the global routing prefix of CS through 6LAR. Moreover, each SN generates an IPv6 address using an IEEE extended unique identifier mechanism or by using the personal area network identity [21].

### 2.2. Threat Model

The Dolev-Yao (DY) model [22] is the threat model used in S6AE. According to the DY model, an intruder can intercept and record the messages exchanged between two communicating entities in 6LoWPAN. Communications among the entities in 6LoWPAN are public in nature. If an adversary has the knowledge about the private key, it can encrypt and decrypt messages and perform unlawful activities, such as modifying or forging the captured messages. The communicating entities, such as SNs and 6LDRs, are considered to be untrusted under the DY model. An adversary can capture an SN due to its hostile environment and can extract the sensitive information stored its memory by employing the power analysis attack. However, CS is a central and vital component in the 6LoWPAN environment, and it cannot be compromised by an adversary.

### 2.3. Preliminaries

#### 2.3.1. Hash Function

A hash function can take a variable size input string, and return the output with a fixed size string. Each hash function must obey the following properties.

- The output of the hash function with two different inputs, *n* and *m*, can never be the same, i.e., H(n)≠H(m).
- It is not possible to compute the input, *z*, from the output of a hash function, H(z), i.e., ${\left(H\left(z\right)\right)}^{-1}\neq z$.

#### 2.3.2. ASCON

**Figure 2:**
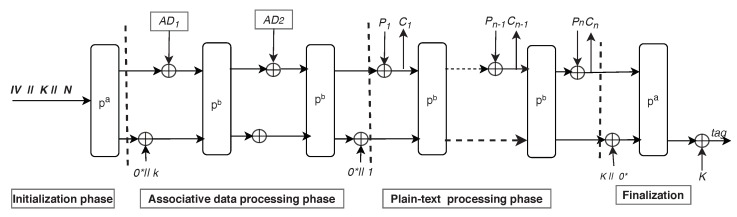
ASCON architecture.

ASCON is an authenticated encryption with associated data scheme [23] that works on the design principle of duplex sponge architecture. Moreover, ASCON is a symmetric, inverse free, single pass, and online block cipher. Broadly speaking, there are two versions of ASCON: (i) ASCON-128 that takes 64 bits data block and generates 64 bits ciphertext along with 128 bits of authentication tag, and (ii) ASCON-128a that takes 128 bits data block and generates 128 bits of ciphertext along with 128 bits of authentication tag. The architecture of ASCON is given in Figure 2, which works under the following four stages [23]. Initialization: In this stage, ASCON computes the initial input to ASCON state by combining the Initialization Vector (IV), nonce, and key. The size of ASCON state is 320 bits. Associated Data (AD) Processing: This stage processes AD that represents the data block to be transmitted in an un-encrypted form, while at the same time ensuring the integrity of the transmitted data block. Plaintext Processing: In this stage, ASCON takes plaintext as an input and generates the ciphertext as output. Finalization: In the final stage, ASCON generates the authentication tag, which ensures the integrity and authenticity of the ciphertext and AD. Furthermore, the substitution and permutation network of ASCON comprises 5-bit S-Box, bit-wise XOR, and rotation operations. Thus, ASCON is suitable for resource-constrained devices, such as embedded systems and radio frequency identifier tags, because of its lightweight property and minimal overheads [24,25,26]. For securing 6LoWPANs, the proposed S6AE scheme in this paper borrows the standard ASCON encryption design, which provides confidentiality and authenticity of data simultaneously. Additionally, S6AE employs SHA-256 hash function, and bit-wise XOR operations to achieve AKE in 6LoWPANs.

## 3. The Proposed S6AE Scheme

S6AE verifies the legitimacy of SNs at the CS, and validates the integrity and authenticity of messages exchanged between SNs and the CS in 6LoWPANs. In S6AE, after verifying the authenticity of SNs, CS and SNs establish secret keys using ASCON as the encryption scheme. SHA-256 is used to generate unique output strings by using the S6AE secret parameters, and bit-wise XOR operations are used to reduce the computational and storage costs. S6AE consists of the registration phase, the AKE phase, and the handover phase. It is necessary for a static or mobile SN to execute the first two phases, whereas only a mobile SN requires to execute the handover phase. The notations used in this paper are listed in Table 1.

### 3.1. Sensor Registration Phase

This phase deals with the registration of SN before its deployment in 6LoWPAN. CS performs the following operations to register SNs. It

- calculates the master key $K_{m}$ by computing $K_{m}=H\left(ID_{cs}\|r_{cs}\right)$, where $ID_{cs}$ is the real identity of CS and $r_{cs}$ is a random number. CS divides $K_{m}$ into four equal chunks of 64 bits, namely $K_{m}^{1}$, $K_{m}^{2}$, $K_{m}^{3}$, and $K_{m}^{4}$, and computes $K_{cs}=K_{m}^{1}⊕K_{m}^{2}⊕K_{m}^{3}⊕K_{m}^{4}$, where $K_{cs}$ is a temporary key for CS.
- assigns a unique $ID_{sn}$ of 64 bits for SN.
- picks a key $K_{sn}$ of 64 bits for SN and computes the pseudo-identity $SID_{sn}=ID_{sn}⊕K_{sn}⊕K_{cs}$.
- computes $H_{r}=H(K_{m}\|K_{sn}\left\|ID_{sn}\right)$ and derives security parameter $SP_{1}$ by computing $SP_{1}=H_{r}^{1}⊕H_{r}^{2}⊕H_{r}^{3}⊕H_{r}^{4}$, where $H_{r}^{1}$, $H_{r}^{2}$, $H_{r}^{3}$, and $H_{r}^{4}$ are four equal chunks of 64 bit $H_{r}$.

Finally, CS stores SN related secret information, i.e., {$ID_{sn}$, $SP_{1}$, $K_{sn}$, $K_{cs}$, MACsn} into its database and {$ID_{sn}$, $SP_{1}$, $SID_{sn}$, $K_{sn}$, MACcs} in the memory of SN while making use of a secure channel. CS also stores $SID_{sn}$ into 6LDR memory through a secure channel.

### 3.2. Sponge State Generation

The initialization phase $S_{k}$ of ASCON consists of 320 bits, known as initialization states $S_{k}$. In the proposed scheme, $S_{k}$ can be derived as follows. SN

- generates a random number $R_{1}$ of 64 bits and time stamp $T_{sn}$ of 32 bits,
- computes $IV_{sn}=R_{1}\|SID_{sn}$, where $IV_{sn}$ is an initialization vector for SN,
- computes $H_{s}=H(ID_{sn}\|SID_{sn}\|SID_{ldr}\left\|T_{sn}\right)$ and derives $S_{k}=IV_{sn}\|H_{s}^{24}$, where $H_{s}^{24}$ is the first 24 bytes of $H_{s}$. The size of $S_{k}$ is 320 bits ($H_{s}^{24}$ = 24 bytes + $IV_{sn}$ = 16 bytes), which is served as input to the encryption algorithm during the initialization phase.

### 3.3. Associative Data Generation

The proposed S6AE scheme generates AD while incorporating the compressed IPv6 and User Datagram Protocol (UDP) headers [8,21]. The header size is 10 bytes after compression. The subsequent Immutable Fields (IF) are used to generate AD. This includes parameters such as dispatch, internet protocol header compression, context identifier, next header compression, destination interface identifier, UDP Ports, UDP Checksum, Global routing prefix of 6LoWPAN (G6), and Global routing prefix of CS (GC). CS stores the MAC of SN and SN stores the MAC of CS. Moreover, the hop limit parameter is mutable, which is not incorporated in AD generation. The following operations are performed to generate AD.

- SN computes $H_{ad}=H(IF_{sn}\|G6\|GC\|MAC_{sn})$. It then divides $H_{ad}$ into two equal parts, i.e., $H_{ad}^{1}$ and $H_{ad}^{2}$ each of 128 bits.
- SN computes $AD=H_{ad}^{1}⊕H_{ad}^{2}$ and divides AD into two equal parts, i.e., $AD_{1}$ and $AD_{2}$, each of 64 bits.
- The encryption algorithm takes $AD_{1}$ and $AD_{2}$ as the inputs at the associative data processing phase to preserve their integrity.

**Remark** **1.** *During the registration process of SN, CS stores the credential information in SN’s memory. Based on this secret, S6AE computes $S_{k}$, which is the initialization phase of the encryption algorithm as discussed in Section 3.2. The unencrypted information, such as IPv6/UDP information, is used for the associative data processing phase of the encryption algorithm, which is described in Section 3.3. The same process is repeated at the receiver side for decryption.*

### 3.4. Authentication and Key Exchange

In this phase, SN achieves the anonymous authentication and key agreement with CS via the intermediate nodes, 6LDR and 6LAR. After establishing a secret key, SN and CS can exchange data securely. S6AE exchanges four messages to accomplish the authentication process. The detail of the messages exchanged in the proposed scheme is given below.

#### 3.4.1. Step AKE-1

SN generates a random number $R_{s1}$ of 64 bits and timestamp $T_{sn}$ of 32 bits for computing $X=ID_{sn}⊕R_{s1}⊕SP_{1}$, and $Y=ID_{sn}⊕R_{s1}$, where the sizes of *X* and *Y* are 64 bits. The encryption algorithm takes $S_{k}$ as shared secret inputs during the initialization phase, $\langle AD_{1},AD_{2}\rangle$ at the associative data processing phase, which is computed in Section 3.3, 〈X‖Y〉 at the plaintext processing phase, and produces ciphertext $C_{1}=E_{S_{k}}\left\{AD_{1},AD_{2},\langle X\|Y\rangle \right\}$ and $Tag_{sn}$ that is generated automatically by ASCON. $C_{1}$ ensures the confidentiality of the plaintext 〈X‖Y〉. The generated $Tag_{sn}$ guarantees the authenticity and integrity of the ciphertext $C_{1}$ at the receiving end. $Tag_{sn}$ provides the same functionality as Message Authentication Code (MAC). SN also computes $Z=SID_{sn}⊕SID_{ldr}$, where $SID_{ldr}$ is the temporary identity of 6LDR. After performing the above operations, SN constructs a message M1:$\langle T_{sn}\|Z\|\langle C_{1}\|Tag_{sn}\rangle \|R_{1}\rangle$ and forwards it to 6LDR to be processed further.

**Remark** **2.** *There are various encryption algorithms, such as the Advanced Encryption Standard (AES), which provides confidentiality features. However, AES does not provide authentication of data. To achieve the required authentication, another algorithm is required, such as the MAC algorithm. Thus, all authenticated encryption schemes can be used to achieve confidentiality and authenticity of the communicated message because these schemes generate ciphertext as well as authentication tag. The authentication tag renders the same functionality as that of the MAC algorithm. This implies that an authenticated encryption scheme provides the same functionality as that of the cumulative AES and MAC functionality. An AKE scheme, which is based on AES, requires another cryptographic algorithm to achieve the authenticity of messages.*

*The main idea here is to use ASCON to achieve the cumulative functionality of AES + MAC by using a single algorithm (i.e., ASCON), which generates its own MAC to be validated at the destination. To check the integrity of transmitted messages, we do not to employ any other MAC. In this way, we are able to reduce the computational cost, as shall be demonstrated in the performance evaluation section.*

#### 3.4.2. Step AKE-2

After receiving M1 from SN, 6LDR picks out *Z* from the received message and computes $SID_{r}=Z⊕SID_{sn}$. 6LDR compares $SID_{r}$ with the stored $SID_{ldr}$ in its memory. If the contents of both the $SID_{r}$ and $SID_{ldr}$ are the same, 6LDR appends its $SID_{ldr}$ with the received M1 for generating and forwarding the new message M2:$\langle SID_{ldr}\|M1\rangle$ to 6LAR. Contrarily, 6LDR aborts the AKE process and sends an error message back to SN.

#### 3.4.3. Step AKE-3

6LAR receives the newly generated M2 from 6LDR and checks $SID_{ldr}$ in the current list of the registered devices. If 6LAR does not find $SID_{ldr}$ in the list, it will abort the AKE process and add unverified $SID_{ldr}$ in the blacklist. On the contrary, upon successful verification of the $SID_{ldr}$ for M2, 6LAR picks a timestamp $T_{lar}$ and computes $H_{lar}=H(M2\|SID_{lar}\|T_{lar}\left\|K_{lar}\right)$, where $K_{lar}$ is the pre-shared key between 6LAR and CS, and $SID_{lar}$ is the temporary identity of 6LAR. 6LAR then generates and forwards message M3:$\langle SID_{lar}\|T_{lar}\|M2\|H_{lar}\rangle$ to CS for further processing.

#### 3.4.4. Step AKE-4

Upon receiving M3 from 6LAR, CS retrieves secret information related to 6LAR, such as a $K_{lar}$ using $SID_{lar}$. CS also checks the validity of $T_{lar}$ by verifying if M3 is received within the maximum transmission delay ($T_{d}$) limit by computing $T_{d}\geq T^{r}-T_{lar}$, where $T^{r}$ is the received timestamp of M3. To verify the integrity of M3, CS computes $H_{lar}^{{}^{\prime }}=H(M2\|SID_{lar}\|T_{lar}\left\|K_{lar}\right)$. If the computed $H_{lar}^{{}^{\prime }}$ and the received $H_{lar}$ are not identical, CS aborts the AKE process and adds 6LAR to the current list of fake devices. After checking the integrity of M3, CS retrieves M2 from M3, and checks if the condition $T_{d}\geq T^{r}-T_{sn}$ holds. If the condition does not hold, then CS rejects M2. Moreover, CS also checks whether a valid $SID_{ldr}$ exists in the current list of 6LDR devices. On successful verification of $SID_{ldr}$, CS picks Z from M2, derives $SID_{sn}$ by computing $SID_{ldr}⊕Z$, and checks if $SID_{sn}$ exits in its database. After the verification of the $SID_{sn}$, CS retrieves the information stored in its database, such as $ID_{sn}$, $K_{cs}$, $K_{sn}$, and $SP_{1}$.

#### 3.4.5. Step AKE-5

CS generates $IV_{cs}$ by concatenating $R_{1}$ with $SID_{sn}$, which are attached with the received M2. CS also computes $H_{s}^{{}^{\prime }}=H(ID_{sn}\|SID_{sn}\|SID_{ldr}\left\|T_{sn}\right)$ to derive $S_{k}^{{}^{\prime }}$. It is important to mention here that 320 bits of $S_{k}^{{}^{\prime }}$ is the concatenation of $IV_{cs}$ and $H_{s}^{{}^{\prime }24}$, i.e., $S_{k}^{{}^{\prime }}=IV_{cs}\|H_{s}^{{}^{\prime }24}$, where $H_{s}^{{}^{\prime }24}$ are the first 24 bytes of the $H_{s}^{{}^{\prime }}$ (which is of 32 byte. The size of $S_{k}^{{}^{\prime }}$ is 40 bytes. Moreover, CS determines AD by using the received header information and the stored $MAC_{sn}^{}$ in CS’s database by computing $H_{ad}^{{}^{\prime }}=H(IF_{sn}\|G6^{}\|GC^{}\left\|MAC_{sn}^{}\right)$, $AD_{x}=H_{ad}^{{}^{\prime }1}⊕H_{ad}^{{}^{\prime }2}$ and divides $AD_{x}$ into two parts, i.e., $AD_{1}^{{}^{\prime }}$ and $AD_{2}^{{}^{\prime }}$. AD is the input to the encryption algorithm and its purpose is to ensure the integrity of header information. The detailed process of computing AD is given in the Section 3.3. In addition, CS performs the decryption operation $D_{s_{k}^{{}^{\prime }}}\left\{\langle AD_{1}^{{}^{\prime }},AD_{2}^{{}^{\prime }},C_{1}\rangle \right\}$, where $S_{s_{k}}^{{}^{\prime }}$ is the input at the initialization phase, $AD_{1}^{{}^{\prime }}$ and $AD_{2}^{{}^{\prime }}$ are the inputs at associative data processing phase, and $C_{1}$ is the input at the ciphertext processing phase, as shown in Figure 2. Moreover, the decryption algorithm generates $Tag_{g}$ before extracting the plaintext information. ASCON generates the authentication tag automatically after processing AD and ciphertext. Then CS checks the condition $Tag_{sn}=Tag_{g}$, where $Tag_{sn}$ is received with $M_{1}$. An inverse free authenticated encryption scheme generates the same authentication tag during the encryption and decryption process, if there is no modification in AD and ciphertext. However, if there is any modification in the communicated message, the generated authentication tag will be different, which causes the failure of authentication process in the proposed AKE. If the condition holds, decryption process will reveal the plaintext information. Otherwise, CS will abort the AKE process. The revealed plaintext, after the decryption of $C_{1}$, includes *X* and *Y*. CS picks the retrieved $ID_{sn}^{}$ and performs $ID_{sn}^{}⊕Y$ operation to determine $R_{s1}$ for computing $SP_{1}^{{}^{\prime }}=ID_{sn}^{}⊕R_{s1}⊕X$. Furthermore, in order to check the legitimacy of SN, CS checks the condition $SP_{1}=SP_{1}^{{}^{\prime }}$. If the condition holds, CS registers SN as a legitimate device, otherwise, CS will abort the AKE process.

#### 3.4.6. Step AKE-6

After verifying the legitimacy of SN, CS picks timestamps $T_{s}$ of 32 bits. CS picks three random numbers $R_{s2}$, $R_{2}$, and $R_{n}$ each of 64 bits. CS then computes $H_{r}^{{}^{\prime \prime }}=H(K_{cs}\|R_{n}\left\|ID_{sn}\right)$ and calculates a new security parameter $SP_{1}^{n}$ by computing $SP_{1}^{n}=H_{r}^{{}^{\prime \prime }1}⊕H_{r}^{{}^{\prime \prime }2}⊕H_{r}^{{}^{\prime \prime }3}⊕H_{r}^{{}^{\prime \prime }4}$, where $H_{r}^{{}^{\prime \prime }1}$$H_{r}^{{}^{\prime \prime }2}$$H_{r}^{{}^{\prime \prime }3}$$H_{r}^{{}^{\prime \prime }4}$ are four equal chunks of $H_{r}^{{}^{\prime \prime }}$ each of 64 bits. CS calculates $Y_{1}=R_{n}⊕K_{cs}$, $X_{1}=Y_{1}⊕R_{s1}$, and $IV_{cs}^{{}^{\prime }}=R_{2}\|X_{1}$, where $IV_{cs}^{{}^{\prime }}$ is the initialization vector at CS and $R_{2}$ is the random number of 64 bits. To generate $S_{k}^{{}^{\prime \prime }}$, CS computes $H_{s}^{{}^{\prime \prime }}=H(ID_{sn}\|R_{s1}\|T_{s}\|T_{exp}\left\|Y_{1}\right)$, where the size of $H_{s}^{{}^{\prime \prime }}$ is 256 bits and calculates $S_{k}^{{}^{\prime \prime }}=H_{s}^{{}^{\prime \prime }24}\|IV_{cs}$, where $H_{s}^{{}^{\prime \prime }24}$ are the first 24 bytes of $H_{s}^{{}^{\prime \prime }}$. Next, CS calculates AD by computing $H_{ad}^{{}^{\prime \prime }}=H(IF_{cs}\|G6\|GC\|MAC_{cs}^{})$, $AD_{x1}=H_{ad}^{{}^{\prime \prime }1}⊕H_{ad}^{{}^{\prime \prime }2}$ and divides $AD_{x1}$ into two parts, i.e., $AD_{1}^{{}^{\prime \prime }}$ and $AD_{2}^{{}^{\prime \prime }}$. For secure communication in future, CS computes a session key $K_{se}$ by calculating $K_{se}=H(ID_{sn}\|Y_{1}\|SP_{1}^{n}\|R_{s1}\left\|R_{s2}\right)$. Moreover, for secure handover from one domain to another domain as shown in Figure 1, CS calculates a unique ticket $T_{ic}^{sn}$ for SN by computing $T_{ic}^{sn}=ID_{sn}⊕R_{s2}⊕R_{s1}\left\|Y_{1}⊕SP_{1}^{n}\right)$. SN will make use of the generated $T_{ic}^{sn}$ during the handover process. CS also picks $T_{ic}^{sn}$’s expiry time $T_{exp}$ (32 bits). In addition, the encryption algorithm takes into account $S_{k}^{{}^{\prime \prime }}$ during the initialization phase, $AD_{1}^{{}^{\prime \prime }}$ and $AD_{2}^{{}^{\prime \prime }}$ during the associative data processing phase, and $\langle SP_{1}^{n}\|R_{s2}\rangle$ during the plaintext information processing phase, in order to generate $C_{2}=E_{S_{k}^{{}^{\prime \prime }}}\left\{AD_{1}^{{}^{\prime \prime }},AD_{2}^{{}^{\prime \prime }},\langle SP_{1}^{n}\|R_{s2}\rangle \right\}$ and $Tag_{cs}$. Moreover, CS constructs the message M4:$\langle T_{cs}\|T_{exp}\|X_{1}\|\langle C_{2}\|Tag_{cs}\rangle \|R_{2}\rangle$, and forwards it to 6LAR. 6LAR and 6LDR simply relay M4 to SN. Furthermore, CS stores the parameters $\{ID_{sn}$, $SP_{1}^{}$, $SP_{1}^{n}$, $K_{cs}$, $T_{ic}^{sn}$, $T_{exp}$} in its memory.

#### 3.4.7. Step AKE-7

**Figure 3:**
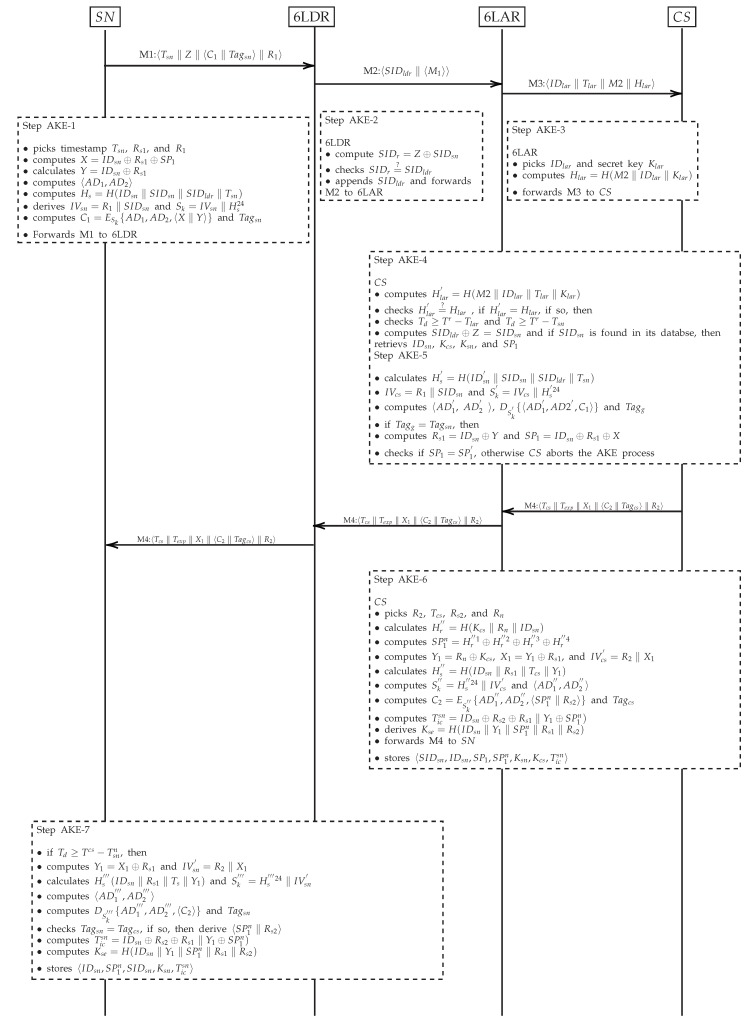
S6AE authentication and key establishment phase.

After receiving M4, SN checks the validity of timestamp $T_{s}$ by checking the condition $T_{d}\geq T^{r}-T_{sn}$, where $T_{d}$ is the maximum allowed time TD and $T^{r}$ is the period in which M4 is received. Significantly, SN will reject M4 if $T_{s}$ exceeds the maximum allowed delay. SN picks $R_{2}$, $X_{1}$ from the received M4 and calculates $IV_{sn}^{{}^{\prime }}=R_{2}\|X_{1}$. SN also computes $Y_{1}=R_{s1}⊕X_{1}$, $H_{s}^{{}^{\prime \prime \prime }}=H(ID_{sn}\|R_{s1}\|T_{s}\left\|Y_{1}\right)$ and $S_{k}^{{}^{\prime \prime \prime }}=H_{s}^{{}^{\prime \prime \prime }24}\|IV_{sn}^{{}^{\prime }}$, where $H_{s}^{{}^{\prime \prime \prime }24}$ is the first 24 bytes of $H_{s}^{{}^{\prime \prime \prime }}$. Next, SN calculates AD by computing $H_{ad}^{{}^{\prime \prime \prime }}=H(IF_{cs}\|G6\|GC\|MAC_{cs})$, $AD_{x2}=H_{ad}^{{}^{\prime \prime \prime }1}⊕H_{ad}^{{}^{\prime \prime \prime }2}$ and divides $AD_{x2}$ into two parts, i.e., $AD_{1}^{{}^{\prime \prime \prime }}$ and $AD_{2}^{{}^{\prime \prime \prime }}$. The decryption algorithm takes $S_{k}^{{}^{\prime \prime \prime }}$ as the input during the initialization phase, $AD_{1}^{{}^{\prime \prime \prime }}$ and $AD_{2}^{{}^{\prime \prime \prime }}$ during the associative data processing phase, $C_{2}$ during the ciphertext processing phase, and performs the decryption operation $D_{S_{k}^{{}^{\prime \prime \prime }}}\left\{AD_{1}^{{}^{\prime \prime \prime }},AD_{2}^{{}^{\prime \prime \prime }},C_{2}\right\}$, to generate $Tag_{sn}^{{}^{\prime }}$. In the final step, SN checks the condition $Tag_{cs}=Tag_{sn}^{{}^{\prime }}$. If the condition holds then decryption algorithm will reveal the plaintext information, i.e., $\langle SP_{1}^{n}\|R_{s2}$〉. Additionally, SN computes the session key $K_{se}$ by computing $K_{se}=H(ID_{sn}\|Y_{1}\|SP_{1}^{n}\|R_{s1}\left\|R_{s2}\right)$ to secure future communications with CS. In addition, SN calculates a unique ticket $T_{ic}^{sn}=ID_{sn}⊕R_{s2}⊕R_{s1}\left\|Y_{1}⊕SP_{1}^{n}\right)$, which will be used during the handover process. Finally, SN stores the parameters {$ID_{sn}$, $SP_{1}^{n}$, $SID_{sn}^{}$, $K_{sn}$, $T_{ic}^{sn}$, $T_{exp}$} in its memory. The AKE phase of the proposed scheme is summarized in Figure 3.

### 3.5. Handover Phase

In the proposed scheme, a sensor node can move from network Domain-1 to another Domain-2, as shown in Figure 1. Hence, it is essential to verify the authenticity of a roaming SN with minimal overhead complexity. Importantly, SN utilizes the ticket $T_{ic}^{sn}$, generated during the AKE phase, to accomplish fast authentication. More specifically, SN performs the following operations during the handover process.

#### 3.5.1. Step HP-1

When an SN moves from the communication range of 6LDR1 in Domain-1 to the communication range of 6LDR2 in Domain-2, SN sends a handover request to 6LDR2. SN checks $T_{exp}$ of $T_{ic}^{sn}$, which is stored in $SN^{\prime }$s memory. If $T_{ic}^{sn}$ is not expired then SN picks the timestamp $T_{h}$ and computes $H_{h}=H(T_{ic}^{sn}\|T_{h}\left\|SID_{sn}\right)$. SN then constructs a message $M_{h1}$:$\langle SID_{sn}\|T_{h}\|T_{ic}^{sn}\|H_{h}\rangle$ and forwards $M_{h1}$ to 6LDR2. 6LDR2 checks if $T_{h}$ is fresh or not. To check integrity of $M_{h1}$, 6LDR2 computes $H_{h2}=H(T_{ic}^{sn}\|T_{h}\left\|SID_{sn}\right)$ and checks the condition $H_{h2}=H_{h}$. If the condition holds, 6LDR2 stores $SID_{sn}$ in its memory and forwards $M_{h1}$ to CS. Contrarily, CS aborts the handover process and adds $SID_{sn}$ into blacklist in its database. After receiving $M_{h1}$, CS computes $H_{h3}=H(T_{ic}^{sn}\|T_{h}\left\|SID_{sn}\right)$ and checks the condition $H_{h3}=H_{h}$. If the condition holds, CS checks if $SID_{sn}$ exists in its database and verifies the condition $T_{ic}^{sn}=T_{ic}^{sn}$. If the condition holds, CS continues the handover process, otherwise CS marks $ID_{sn}$ as a compromised node and broadcasts $ID_{sn}$ in the network. CS also sends a message to 6LDR1 to delete $SID_{sn}$ from its memory. 6LDR1 sends an acknowledgment to CS. $T_{ic}^{sn}$ is the stored ticket at SN and CS.

#### 3.5.2. Step HP-2

CS picks two random numbers $R_{n}$, $R_{1}$ each of 64 bits, and timestamps $T_{exp}^{n}$ and $T_{h1}$ each of 32 bits. It also computes $S_{k}^{h}=\left(K_{es}\|R_{h}⊕ID_{sn}\right)$ and $P=R_{n}⊕SP_{1}^{n}$, where the size $SP_{1}^{n}$ is 64 bits. CS calculates $C_{h}=E_{S_{k}^{h}}\left(P\right\|T_{exp}^{n}\left\|T_{h1}\right)$ and $Tag_{cs}$ by using the encryption algorithm. The $Tag_{cs}$ ensures the authenticity of the transmitted information. It also computes the new session key as $K_{se}^{n}=H(ID_{sn}\|R_{n}\left\|K_{se}\right)$. CS constructs a message $M_{h2}$:$\langle SID_{sn}\|C_{h}\|Tag_{cs}\rangle$ and forwards $M_{h2}$ to 6LDR2. Upon receiving $M_{h2}$, 6LDR2 looks up $SID_{sn}$ in 6LDR2’s memory. If $SID_{sn}$ exists in the memory of 6LDR2, 6LDR2 forwards $M_{h2}$ to SN.

#### 3.5.3. Step HP-3

**Figure 4:**
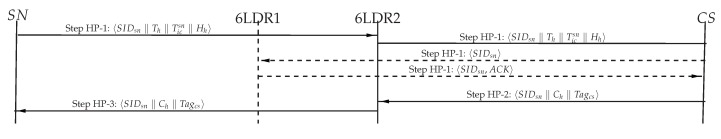
The S6AE handover process.

After receiving the message $M_{h1}$ from CS, SN performs the decryption using $D_{S_{k}^{{}^{\prime }h}}\left\{C_{h}\right\}$, where $S_{k}^{{}^{\prime }h}=\left(K_{es}\|R_{h}⊕ID_{sn}\right)$. The decryption process reveals the plaintext, which is $\left(P\|T_{exp}^{n}\|T_{h1}\right)$ and also it generates the $Tag_{sn}$. SN checks the condition $T_{d}\geq T^{r}-T_{h}$. If the condition holds then SN considers $M_{h1}$ valid, otherwise it rejects $M_{h1}$. $T_{exp}^{n}$ indicates new expiry time of the $T_{ic}^{sn}$. SN checks the condition $Tag_{sn}=Tag_{cs}$. If the condition holds, then SN computes $R_{n}^{{}^{\prime }}=SP_{1}^{n}⊕P$ and $SP_{1}^{{}^{\prime }n}=P⊕R_{n}^{{}^{\prime }}$. Authentication will be successful if the stored $SP_{1}^{n}$ and the computed $SP_{1}^{{}^{\prime }n}$ are the same. SN generates a symmetric key between SN and CS by computing $K_{se}^{n}=H(ID_{sn}\|R_{n}\left\|K_{se}\right)$. Finally, SN replaces the stored session key $K_{se}$ with the new session key $K_{se}^{n}$ in the memory and updates the expiry time $T_{exp}^{n}$ in the tuple {$ID_{sn}$, $SP_{1}^{n}$, $SID_{sn}$, $K_{se}^{n}$, $T_{ic}^{sn}$, $T_{exp}^{n}$}. Figure 4 shows the message exchange during the handover phase.

## 4. Security Analysis

This section analyzes the security properties of our proposed S6AE scheme in three different phases. In the first phase, the characteristics and capabilities of the S6AE scheme against malicious attacks are described. In the second phase, BAN logic is incorporated to show the logical correctness of the S6AE scheme. In the final phase, AVISPA tool is used for automatically verifying the security properties of the proposed strategy.

### 4.1. Informal Security Analysis

#### 4.1.1. Header Verification

Header Verification (HV) is an effective mechanism to mitigate the replay and Denial-of-Service (DoS) attacks. In the proposed scheme, to provide IPv6/UDP header verification, SN computes $H_{ad}=H(IF\|G6\|GC\|MAC_{sn})$, $AD=H_{ad}^{1}⊕H_{ad}^{2}$, where $H_{ad}^{1}$ and $H_{ad}^{2}$ are the two equal chunks each of 128 bits of $H_{ad}$. SN divides AD into two equal parts, i.e., $AD_{1}$ and $AD_{2}$ each of 64 bits, which are the inputs at the associative data processing phase of the encryption algorithm. After receiving the message M1, CS computes $\langle AD_{1}^{{}^{\prime }},AD_{2}^{{}^{\prime }}\rangle$ and the decryption algorithm takes $\langle AD_{1}^{{}^{\prime }},AD_{2}^{{}^{\prime }}\rangle$ at the associative data processing phase. If there is no modification in the IPv6/UDP header, then the condition $Tag_{sn}=Tag_{g}$ will hold. This condition will not hold if an adversary modifies the IPv6/UDP header during the AKE process. The same procedure holds for the message transmitted from CS to SN. In this way, the proposed scheme ensures IPv6/UDP header integrity (origin verification).

**Remark** **3.** *In this paper, HV means verification of the IPv6 header at the receiving end. We achieve HV by generating AD through the Hash function SHA-256, as discussed in Section 3.3. If A tries to modify the the IPv6 header, the generated authentication tag will not match the authentication tag attached with the received message.*

#### 4.1.2. DoS Attack

By a DoS attack, an attacker can perform malicious activities and prevent a legal user from accessing the network resources [11]. A DoS attack can degrade the performance of the network. An IP spoofing attack is used to launch the DoS attack in the network by generating a large amount of data packet with fake IP addresses. S6AE can provide protection against the IP spoofing attack by ensuring the integrity of the IPv6 header. To perform a DoS attack, an adversary needs to calculate $H_{s}^{{}^{\prime }A}=H(ID_{sn}^{{}^{\prime }A}\|SID_{sn}^{A}\|SID_{ldr}^{A}\left\|T_{sn}^{A}\right)$, $IV_{cs}=R_{1}\|SID_{sn}$, $S_{k}^{{}^{\prime }A}=IV_{cs}^{A}\|H_{s}^{{}^{\prime }24A}$, 〈$AD_{1}^{{}^{\prime }}$, $AD_{2}^{{}^{\prime }}$〉, $D_{S_{k}^{{}^{\prime }}}\left\{\langle AD_{1}^{{}^{\prime }},AD2^{{}^{\prime }A},C_{1}^{A}\rangle \right\}$, and $Tag_{g}^{A}$. Then A checks the condition $Tag_{g}^{A}\overset{}{=}Tag_{sn}$. The condition $Tag_{g}^{A}\overset{}{=}Tag_{sn}$ will not hold after capturing the IPv6/UDP header information because A requires the parameters, such as $ID_{sn}$, $SP_{}$, and $K_{sn}$, which are secrets to SN and CS. Thus, S6AE can protect against DoS attacks.

#### 4.1.3. Replay Attack

A sort of network attack in which attacker wiretaps or captures the valid transmitted data and retransmits the seized data in the network for harmful intention [27]. During the authentication process (Section 3.4), all the transmitted messages M1:$\langle T_{sn}\|Z\|\langle C_{1}\|Tag_{sn}\rangle \|R_{1}\rangle$, M2:$\langle SID_{ldr}\|M1\rangle$, M3:$\langle ID_{lar}\|T_{lar}\|M2\|H_{lar}\rangle$, and M4:$\langle T_{cs}\|T_{exp}\|X_{1}\|\langle C_{2}\|Tag_{cs}\rangle \|R_{2}\rangle$ include timestamps, and random numbers. The verification of the timestamps, such as $T_{sn}$, $T_{cs}$, and $T_{lar}$, ensure the freshness of the received message. Usually, $T_{d}$ is very small. Therefore, within $T_{d}$, the probability of replaying M1, M2, M3, and M4 for adversary A is negligible. A similar situation holds for the handover phase messages. S6AE also prevents the replay attack by ensuring the IPv6/UDP header integrity. Any modification in the IPv6/UDP header during the transmission of a message through the public Internet makes the decryption and authentication unsuccessful at the respective communicating entities, such as CS and SN. Hence, S6AE is secure against the replay attacks.

#### 4.1.4. Man-in-the-Middle (MITM) Attack

MITM is an action of an intruder in which the intruder somehow conjoins the communication between the two communicating network nodes while both the nodes believe that they are communicating directly [28]. Let an adversary A captures all the transmitted messages M1, M2, M3, and M4 during the communication between SN and CS. Suppose A attempts to forge M1 to generate a valid message to force CS to believe that the forged message is from an authentic source. For this purpose, A needs to guess the real identity $ID_{sn}$ of SN, which is an infeasible task for A. Therefore, it not possible for A to generate a bogus message M1. A similar condition holds for all other transmitted messages. This clearly indicates that S6AE is protected against MITM attack.

#### 4.1.5. Sensor Impersonation Attack

By using an impersonation attack, the attacker can impersonate as an authentic SN to perform malicious activities in the network [11]. To execute this attacks, an adversary A picks the current timestamp $T_{sn}^{{}^{\prime }}$, $ID_{sn}^{{}^{\prime }}$ and random number $R_{s1}^{{}^{\prime }}$ and then attempts to transmit the message M1 to CS on behalf of SN. However, to construct a legitimate $M1^{{}^{\prime }}$, A must know the real identity $ID_{sn}$ of SN, $R_{s1}$ and $SP_{1}$. Without knowing these parameters, it is hard for A to generate valid $S_{k}=IV_{sn}\|H_{s}^{24}$ and $C_{1}$. For A, it is computationally hard to generate $ID_{sn}$, $SP_{1}$ and $R_{s1}$. Therefore, A cannot generate a legitimate M1 and, thus, the proposed scheme provides protection against the impersonation attack.

#### 4.1.6. Server Impersonation Attack

In this attack, adversary A can send M4 to SN on behalf of CS. To compute a valid $S_{k}^{{}^{\prime \prime }}=H_{s}^{{}^{\prime \prime }24}\|IV_{cs}^{{}^{\prime }}$ and $C_{2}$, it is necessary for A to know the secret parameters $ID_{sn}$, $K_{cs}$, $R_{n}$, and $R_{s1}$. However, for A, it is computationally hard to generate these parameters, which are known only to CS. Therefore, S6AE can mitigate CS impersonation attacks.

#### 4.1.7. Identity Privacy Preservation

Normally, SN utilizes the pseudo-identity $SID_{sn}$ during the transmission of the authentication messages, which is computed as $SID_{sn}=ID_{sn}⊕K_{sn}⊕K_{cs}$, where all the parameters are secret to CS and SN. Therefore, it is hard for A to generate $SID_{sn}$ without knowing these parameters. This demonstrates that the proposed scheme ensures the identity privacy of SN.

#### 4.1.8. Unlinkability/Anonymity

S6AE renders the unlinkable and anonymous session during the AKE process. Each time when a new session starts, SN picks a fresh random number $R_{1}$ and generates an $IV_{sn}=R_{1}\|SID_{sn}$. The newly generated $IV_{sn}$ is the input to the initialization phase of the encryption algorithm. The encryption algorithm produces different ciphertext each time even with the same secret parameters $SP_{1}$, $R_{s1}$, and $ID_{sn}$. The ciphertext also includes another fresh random number $R_{s1}$, which in turn enhances the randomness of the ciphertext. Therefore, it is hard for an adversary to correlate the two sessions form the same node. S6AE is untraceable, and it is not possible for an attacker to create a link between two different AKE processes. Since each AKE session utilizes a new $SID_{sn}$, this makes the AKE session anonymous. Hence, S6AE ensures unlinkability and anonymity during the AKE process.

#### 4.1.9. Sybil Attack

In a Sybil attack, the adversary can generate multiple counterfeit identities of real nodes. S6AE can prevent the Sybil attack because each SN in the network authenticates itself with CS [11]. If CS discover any duplicate $ID_{sn}$ of an SN during the AKE process in the database, then CS considers that particular ID as a compromised node. CS adds these IDs to the blacklist and forwards the list to 6LDR1 and 6LDR2, which in turn broadcast these IDs in the network. Thus, S6AE protects against the Sybil attack.

#### 4.1.10. Forward/Backward Secrecy

Forward/backward secrecy means that if an adversary reveals the current session key, it does not enable an intruder to compromise the privacy of the past and future session keys [11]. S6AE determines session key by computing $K_{se}=H(ID_{sn}\|Y_{1}\|SP_{1}^{n}\|R_{s1}\left\|R_{s2}\right)$ for each AKE session. A new AKE process establishes a session key by incorporating fresh parameters, such as $Y_{1}$, $SP_{1}^{n}$, $R_{s1}$, and $R_{s2}$. If an adversary A breaches the security of the current session key $K_{se}$, it does not allow A to compromise the future session key. Therefore, it is hard for an adversary to construct the past or future session keys.

#### 4.1.11. Ephemeral Secret Leakage (ESL) Attack

Pre-computed Ephemeral Secrets (ES), which are stored in insecure memory, can be compromised by A. By using these compromised ES (short term) and long term parameters, A can breach the session key security. Such types of attacks are known as ESL [29]. In S6AE, SN and CS establish a secret session key $K_{se}$ during the AKE process for the future secure communication. The established session key $K_{se}=H(ID_{sn}\|Y_{1}\|SP_{1}^{n}\|R_{s1}\left\|R_{s2}\right)$ incorporates ephemeral terms, such as $R_{s1}$, $R_{s2}$, and long terms, such as $ID_{sn}$. If A compromises the ephemeral terms $R_{s1}$ and $R_{s2}$, A still requires the long term $SID_{sn}$ to breach the the security of the session key $K_{se}$. To compromise the security of $K_{se}$, A must know the valid long and ephemeral terms, which are hard for A to know. Therefore, the proposed S6AE is resilient to the ESL attack.

### 4.2. Crypt-Analysis Using BAN Logic

The BAN logic [30] is a logic of belief and action. It is a well defined formal method to test the logic correctness of a security protocol and determines the trustfulness of agreement among the participants in the AKE process of S6AE. The BAN logic is employed here to validate the mutual authentication properties of the proposed S6AE scheme as a whole. The notations used in the BAN logic are listed in the Table 2, which are used to describe different inference rules. A list of BAN logic inference rules are listed in Table 3, which are used to determine the goal of the proposed scheme.

#### 4.2.1. Assumptions

S6AE makes the following assumptions at the outset to investigate the AKE properties of our scheme.

AS-1: $SN|\equiv \#\left(T_{sn}\right),\#\left(T_{cs}\right)$
AS-2: $CS|\equiv \#\left(T_{sn}\right),\#\left(T_{cs}\right)$
AS-3: $CS|\equiv ID_{sn}$
AS-4: $CS|\equiv SP_{1}$
AS-5: $SN|\equiv ID_{sn}$
AS-6: $SN|\equiv SP_{1}$
AS-7: $CS|\equiv (CS\overset{ID_{sn}}{↔}SN)$
AS-8: $SN|\equiv (CS\overset{ID_{sn}}{↔}SN)$
AS-9: $SN|\equiv \#(R_{s1})$
AS-10: $CS|\equiv \#(R_{s1})$
AS-11: $SN|\equiv CS|\equiv (SN\overset{ID_{sn}}{↔}CS)$
AS-12: $SN|\equiv CS\Rightarrow (SN\overset{K_{se}}{↔}CS)$
AS-13: $CS|\equiv SN\Rightarrow (SN\overset{SP_{1}}{↔}CS)$
AS-14: $CS|\equiv (CS\overset{SP_{1}}{↔}SN)$
AS-15: $SN|\equiv (SN\overset{SP_{1}}{↔}CS)$

#### 4.2.2. Goals

To verify the AKE process of S6AE, it must achieve the following goals.

G1: $CS|\equiv SN|\equiv (SN\overset{SP_{1}}{↔}CS)$
G2: $CS|\equiv (CS\overset{SP_{1}}{↔}SN)$
G3: $SN|\equiv CS|\equiv (CS\overset{K_{se}}{↔}SN)$
G4: $SN|\equiv (CS\overset{K_{se}}{↔}SN)$

#### 4.2.3. Protocol Idealized Form

The idealized form of the proposed scheme can be expressed as follow.

IF1: SN→CS: $\left(T_{sn},{\left\{SP_{1},R_{s1}\right\}}_{ID_{sn}}\right)$
IF2: CS→SN: $\left(T_{cs},Y_{1},{\left\{SP_{1}^{n},R_{s2},\left(CS\overset{K_{se}}{↔}SN\right)\right\}}_{ID_{sn}}\right)$

#### 4.2.4. Formal Verification

In this phase of the BAN logic, the inference rules, listed in Table 3, are used to determine if S6AE has achieved its security goals.

VF-1: From IF1, AS-7, AS-8, and by applying Message-Meaning-Rules, it is possible to achieve
  (1) $$\frac{CS|\equiv \left(SN\overset{ID_{sn}}{↔}CS\right),CS⊲\left(T_{sn},{\left\{SP_{1},R_{s1}\right\}}_{ID_{sn}}\right)}{CS|\equiv SN|∼(T_{sn},{\left\{SP_{1},R_{s1}\right\}}_{ID_{sn}})}.$$
VF-2: From IF1, AS-2 and by applying Freshness-Rule concludes
  (2) $$\frac{CS|\equiv \#(T_{sn})}{CS|\equiv \#(T_{sn},\left\{SP_{1},R_{s1}\right\})}.$$
VF-3: Using VF-1, VF-2 and by applying the Nonce-Verification-Rule, it is possible to obtain
  (3) $$\frac{CS|\equiv \#\left(T_{sn},\left\{SP_{1},R_{s1}\right\}\right),CS|\equiv SN|∼\left(T_{sn},\left\{SP_{1},R_{s1}\right\}\right)}{CS\equiv SN|\equiv (T_{sn},\left\{SP_{1},R_{s1}\right\})}.$$
VF-4: From VF-3 and by applying the Belief-Rule, the goal G1 can be achieved as
  (4) $$\frac{CS|\equiv SN|\equiv (T_{sn},\left\{SP_{1},R_{s1}\right\})}{CS|\equiv SN|\equiv (SN\overset{SP_{1}}{↔}CS)}.$$
VF-5: The goal G2 can be accomplished by utilizing VF-4, AS-13, and by employing the Jurisdiction-Rule from
  (5) $$\frac{CS|\equiv SN|\Rightarrow \left(SN\overset{SP_{1}}{↔}CS\right),CS|\equiv SN|\equiv \left(SN\overset{SP_{1}}{↔}CS\right)}{CS|\equiv (SN\overset{SP_{1}}{↔}CS)}.$$
VF-6: From IF2, AS-11, and by applying Message-Meaning-Rules, it is possible to derive
  (6) $$\frac{SN|\equiv \left(SN\overset{ID_{sn}}{⇆}CS\right),CS⊲\left(T_{cs},Y_{1},{\left\{SP_{1}^{n},R_{s2},\left(CS\overset{K_{se}}{↔}SN\right)\right\}}_{ID_{sn}}\right)}{SN|\equiv CS|∼(T_{cs},,Y_{1},{\left\{SP_{1}^{n},R_{s2},\left(CS\overset{K_{se}}{↔}SN\right)\right\}}_{ID_{sn}})}.$$
VF-7: By using IF2, AS-1, and utilizing the Freshness-Rule, we get
  (7) $$\frac{SN|\equiv \#(T_{cs})}{SN|\equiv \#(Y_{1},\left\{SP_{1}^{n},R_{s2},\left(CS\overset{K_{se}}{↔}SN\right)\right\})}.$$
VF-8: Using VF-6, VF-7 and by applying the Nonce-Verification-Rule, it is possible to obtain
  (8) $$\frac{SN|\equiv \#(T_{cs},Y_{1},\left\{SP_{1}^{n},R_{s2},\left(CS\overset{K_{se}}{↔}SN\right),\left(CS\overset{ID_{sn}}{↔}SN\right)\right\}),A*}{SN|\equiv CS(Y_{1},\left\{SP_{1}^{n},R_{s2},\left(CS\overset{K_{se}}{↔}SN\right)\right\})},$$
  (9) $$A*=SN|\equiv CS|∼(T_{cs},Y_{1},\left\{SP_{1}^{n},R_{s2},\left(SN\overset{K_{se}}{↔}CS\right)\right\}).$$
VF-9: G3 can be achieved by using VF-8 and by employing the Belief-Rule from
  (10) $$\frac{SN|\equiv CS(T_{cs},Y_{1},\left\{SP_{1}^{n},R_{s2},\left(CS\overset{K_{se}}{↔}SN\right)\right\})}{SN|\equiv CS|\equiv (SN\overset{K_{se}}{↔}CS)}.$$
VF-10: From (11) G4 can be derived by utilizing AS-12 and by employing Jurisdiction-Rule
  (11) $$\frac{CS|\equiv SN|\Rightarrow \left(SN\overset{K_{se}}{↔}CS\right),SN|\equiv CS|\equiv \left(T_{cs},Y_{1},\left\{SP_{1}^{n},R_{s2},\left(CS\overset{K_{se}}{↔}SN\right)\right\}\right)}{SN|\equiv (SN\overset{K_{se}}{↔}CS)}.$$

### 4.3. Crypt-Analysis Using AVISPA

Crypt-analysis of S6AE is conducted using the AVISPA tool [31], which obeys the DY attack model and is commonly used by the research community to examine the capabilities of the security algorithms. AVISPA comprises four back-end models, known as CL-AtSe, TA4SP, OFMC, and SATMC. These back-ends perform various automatic analyses to detect vulnerabilities in the security scheme. It uses perfect cryptography, which means that the adversary cannot derive the messages or plaintext from ciphertext without perceiving the secret key. It uses formal language High-Level Protocol Specification Language (HLPSL) to code a specified security algorithm. A translator known as HLPSL2IF is used to convert the HLPSL code into the Intermediate Form (IF). AVISPA uses four back-end techniques defined in [32] for the automatic analysis and the capabilities of a security algorithm against various attacks. The XOR operation is not supported by SATMC and TA4SP back-end. Therefore, the simulation of S6AE using these two back-ends is not possible.

**Figure 5:**
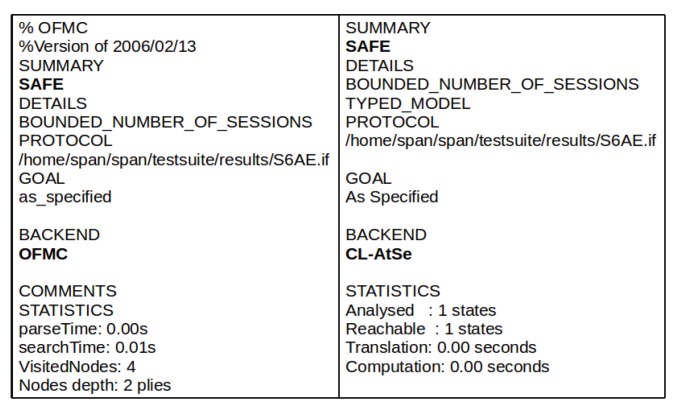
AVISPA OFMC and CL-AtSe back-end simulation results.

Figure 5 shows the Output Format (OF) generated by AVISPA’s OFMC and CL-AtSe back-ends. A generated OF has different sections, including SUMMARY, DETAILS, PROTOCOL, GOAL, BACKEND and STATISTICS, as shown in Figure 5. SUMMARY shows whether a security scheme being tested is safe or unsafe. PROTOCOL describes the HLPSL specification of the scheme in IF. GOALS is the analysis of the goals conducted by AVISPA as specified in HLPSL. BACKEND is used for the backend analysis of the scheme. In S6AE implementation, there are 4 basic roles, i.e., SN, CS, 6LDR, and 6LAR, and two compulsory roles, i.e., environment & goals and session defined in HLPSL. Figure 5 illustrates that the proposed S6AE scheme is secure and protects against MITM and replay attacks.

## 5. Performance Evaluation

This section presents the performance evaluation of S6AE in comparison with eminent 6LoWPAN security schemes, namely, SAKES [16] and EAKES6Lo [17].

S6AE server-side has been implemented in Python 2.7 and each SN is consigned with a unique ID, SID, and SP by CS utilizing a random number generator. Simulations are conducted on a computer with Intel(R) Core(TM) i7-6700 CPU @ 3.40 GHz, Ubuntu (64-bit) and 8-GB RAM. A list of configuration parameters is given in the Table 4.

### 5.1. Security Comparison

The security functionalities of the proposed scheme, compared with the existing security schemes, are given in Table 5. EAKES6Lo and SAKES do not provide any header verification mechanism to mitigates various malicious attacks, such as DoS and replay attacks. EAKES6Lo does not offer identity privacy preservation of the sensor node. However, S6AE is more reliable than other security schemes for 6LoWPAN, as can be seen in Table 5.

### 5.2. Computational Overhead

**Figure 6:**
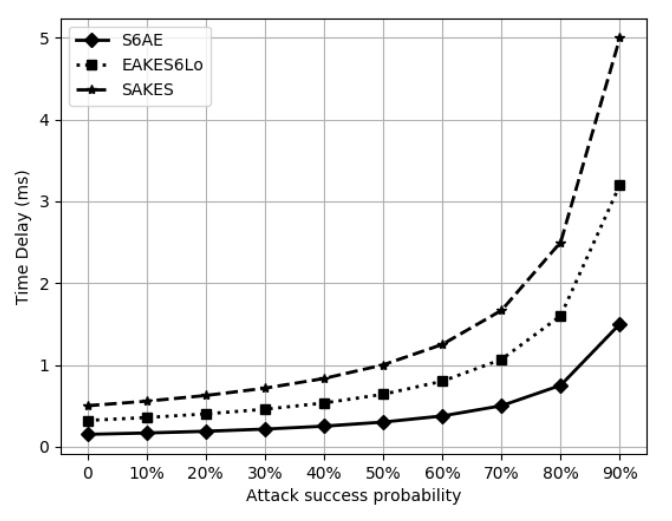
Computational overhead.

The proposed S6AE scheme renders protection against well-known and various covert attacks. However, during the AKE process, many unforeseen attacks, such as a jamming attack, may interfere with the execution of S6AE and may introduce delay during the progress of the AKE process. To estimate the computational overhead, the total execution time delay $T_{d}$ of S6AE can be calculated as

$$T_{d}=\frac{T_{t}}{N_{asp}},$$

where $T_{t}=\sum_{i=1}^{5000}T_{ex}^{i}$ is the total time for 5000 runs, where $T_{ex}^{}$ is the time required for the execution of S6AE and $N_{asp}=5000\times \left(1-attack\;success\;probability\right)$. SAKES is a hybrid security scheme and applies the Diffie-Hellman (DH) key exchange mechanism. Four DH groups provide different levels of security. To achieve the security level of 128-bits, we use the DH group 15 [33]. AES-CTR-128 bits, SHA-256, and ECDSA-160, are the cryptographic operations used by EAKES6Lo during the AKE process. S6AE utilized SHA-256 and ASCON cryptographic operations. The average time consumed by S6AE, SAKES, and EAKES6Lo are 0.417 ms, 1.375 ms, and 0.868 ms, respectively, as shown in Figure 6. Thus, S6AE has the lowest overall computational time.

Furthermore, Table 6 presents the comparison of the computational overheads of SAKES, EAKES6Lo, and S6AE. To compute the computational overheads, this paper considers the average time required for SHA-256, i.e., $T_{sha}=0.0311$ ms, and for the AES-128 is $T_{aes}=0.125$ ms. The time needed for the signature generation/verification is $T_{sg}=5.20$ ms and the time required for ECC public/private key generation is $T_{g}=5.50$ ms. The average time required for ASCON is $T_{ascon}=0.065$ ms (10 MHz) [23,24] and 19.16 ms is the time required for the modular exponentiation (DH). The computational costs of SAKES, EAKES6Lo, and S6AE are $3T_{exp}+8T_{aes}+4T_{sha}\approx 58.6044$ ms, $5T_{aes}+4T_{sha}+2T_{sv}+T_{sg}\approx 17.2494$ ms, and $4T_{ascon}+13T_{sha}+24T_{xor}\approx 0.6643$ ms, respectively. Thus, SAKES and EAKES6Lo are computationally more expensive as compared to the S6AE.

### 5.3. Communication Overhead and Energy Consumption

Optimization of energy consumption is a critical parameter of interest for 6LoWPAN. It is imperative to minimize the transmitted message size to reduce the energy consumption of sensor nodes. 6LAR, CS, and 6LDR are powerful devices with ample energy resources. Therefore, S6AE considers the energy consumption in the wirelessly connected constrained devices, and the energy consumption outside 6LoWPAN is not evaluated. To evaluate the transmission overhead in the proposed scheme, we consider 10 bytes overhead of the compressed form of IPv6/UDP header defined in [21]. The energy consumption during sending and receiving of a single bit is 0.72μJ and 0.81μJ, respectively [34]. The transmission overhead of S6AE is given in Table 7 and energy consumption in Table 8. S6AE has been compared with EAKES6Lo and SAKES. It is observed that S6AE utilizes fewer energy resources.

**Figure 7:**
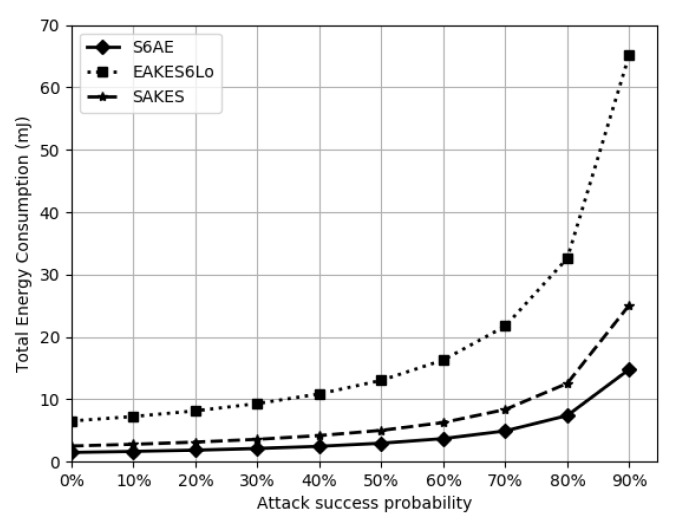
Energy consumption overhead.

The average energy cost for AES encryption/decryption is 9μJ, SHA-256 needs 5.9μJ/byte, ECDSA-160 consumes 6.26 mJ in signature generation, and ASCON requires 0.0207μJ [24]. Total energy cost overhead of the EAKESLo, SAKES, and S6AE are 6.52 mJ, 2.51 mJ, and 1.48 mJ, respectively. If an adversary interrupts the execution of the protocol, it may increase energy consumption. Figure 7 shows the total energy utilization in the presence of jamming attacks.

### 5.4. Storage Overhead Comparison

In the proposed scheme, SN is required to store the tuple {$ID_{sn}$, $SID_{sn}$, $SP_{1}^{n}$, $T_{ic}^{sn}$, $MAC_{cs},$$T_{exp}$ }, which requires (64 + 64 + 64 + 128 + 48 + 32) = 400 bits. CS needs to store the parameters {$SID_{sn}$, $ID_{sn}$, $SP_{1}$, $SP_{1}^{n}$, $T_{ic}^{sn}$, $MAC_{sn}$, $T_{exp}$}, which requires (64 + 64 + 64 + 64 + 128 + 48 + 32) = 464 bits. Table 9 shows the comparison of storage cost of SAKES, EAKES6Lo, and S6AE. It is observed that the proposed scheme requires more storage at the server and less storage at SN as compared to the EAKESLo and needs less storage at SN and CS as compared to SAKES.

### 5.5. Handover Phase Comparison

This section presents the computational and communication overhead during the handover phase. The computational overhead of EAKES6Lo and S6AE are $6T_{aes}+T_{sg}+T_{sv}+6T_{sha}\approx 11.9366$ ms, and $2T_{ascon}+4T_{sha}\approx 0.2544$ ms, respectively, during handover phase. Table 10 shows the communication and computational overheads during the handover phase. The results manifest that the proposed scheme is efficient as compared to the existing schemes.

### 5.6. Discussion

6LoWPANs are at the core of IoT. However, the original 6LoWPAN design does not offer security services, including data confidentiality, integrity and authentication. To address this issue, we have presented an AKE scheme, called S6AE, for 6LoWPANs. For this purpose, we have employed ASCON, which is a lightweight general-purpose encryption algorithm, in conjunction with SHA-256 hash function, to enable the required confidentiality, integrity and authenticity in 6LoWPANs. To the best of our knowledge, ASCON has never been employed in the literature for securing 6LoWPANs.

In S6AE, after verifying the authenticity of SNs, CS and SNs establish secret keys using ASCON as the encryption scheme. ASCON has been employed to achieve data confidentiality and authenticity simultaneously without using a separate MAC. Using AES renders confidentiality, and to achieve the authenticity of the encrypted information it is imperative to use MAC. The main idea in this paper is to use ASCON to achieve the cumulative functionality of AES + MAC by using a single encryption algorithm, i.e., ASCON, which generates its own MAC. To check the integrity of transmitted messages, we do not need to employ any other MAC. In this way, we reduce the computational cost, as compared with the benchmarks.

We use SHA-256 to generate unique output strings by using the S6AE secret parameters. To decrease the communication overhead by means of reducing the message size, the length of the SHA-256 output string must be reduced to 64-bits with minimum computational cost and without compromising performance. For this purpose, we use bit-wise XOR operations. Through BAN logic and AVISPA, we have validated S6AE to be logically complete and offering the required security services in 6LowPANs. We have demonstrated that S6AE reduces the computational and communicational overheads, energy consumption and storage costs, in comparison with the benchmarks.

Results demonstrate that the proposed scheme provides better features in comparison with the benchmarks, namely, EAKES6Lo and SAKES. EAKES6Lo is a hybrid scheme, which uses ECC and AES-CTR and is computationally expensive as compared to S6AE because ECC is resource intensive for the resource constrained 6LoWPANs. Table 6 and Figure 6 show that S6AE requires less resources as compare to EAKES6Lo. Table 5 shows that EAKES6Lo does not provide the identity privacy and header verification. Moreover, SAKES is insecure against the 6LAR gateway compromised attack and does not ensure the header integrity in 6LoWPANs, as shown in Table 5. SAKES employs DH key exchange mechanism and also uses AES as a encryption and decryption scheme, which is computationally expensive in comparison to S6AE, as shown in Table 6 and Figure 6. Table 9 shows that S6AE requires less memory as compared SAKES and EAKES6Lo. Moreover, Table 7 indicates that S6AE is less expensive. Furthermore, S6AE requires less energy resources as compared to the EAKES6Lo and SAKES because S6AE uses lightweight and authenticated encryption that requires less energy and computational resources. In a nutshell, we have found that the implementation of ASCON, in conjunction with SHA-256, in 6LoWPANs is promising to secure communications.

## 6. Conclusions

6LoWPAN is a providential technology having a vital share in IoT and is commonly deployed in a variety of applications. Originally, 6LoWPAN does not provide any security and privacy mechanism. To address this issue, this paper has presented an authentication and key exchange scheme. The proposed scheme establishes a session key after the mutual authentication, which ensures secure communication and prevents an attacker from accessing the transmitted information. The proposed scheme also renders the header verification or origin verification of the message simultaneously without using the IPSec protocol. The employed BAN logic analysis indicates that S6AE is logically complete. Moreover, the security verification using AVISPA illustrates that the proposed scheme is secure against various malicious attacks. Finally, the performance evaluation reveals that, as compared to eminent schemes, S6AE has less communication, computational handover, energy, and storage overheads. As a future work, S6AE can be extended to varying security levels using secure cryptographic algorithms.

## Figures and Tables

**Table 1:** List of notations.

Notation	Description
CS, SN	Central server and 6LoWPAN sensor node
$SID_{sn}$, $SID_{ldr}$,	Pseudo-identities of sensor node and 6LDR, respectively
$ID_{sn}$, $SP_{1}$, $SP_{1}^{n}$	Secret real-identities of 6LoWPAN sensor nodes and secret parameter used in authentication process
$E_{k}\left(x\right)$, $D_{k}\left(x\right)$	Encryption and decryption of message “x” using the secret-key “k”
$\langle Tag_{sn},Tag_{sn}^{{}^{\prime }}\rangle$, $\langle Tag_{g},Tag_{cs}\rangle$	Authentication parameter generated by encryption and decryption algorithm at SN and CS, respectively
$T_{sn}$, $T_{cs}$, $T_{lar}$	Timestamps at SN, CS and 6LAR, respectively.
$\langle IV_{sn},IV_{sn}^{{}^{\prime }}\rangle$, $\langle IV_{cs},IV_{cs}^{{}^{\prime }}\rangle$	Initialization vectors at SN and CS, respectively
$\langle S_{k},S_{k}^{{}^{\prime \prime \prime }}\rangle$, $\langle S_{k}^{{}^{\prime }},S_{k}^{{}^{\prime \prime }}\rangle$	ASCON initialization states at SN and CS, respectively
$S_{k}^{h}$, $S_{k}^{{}^{\prime }h}$	Initialization states at CS and SN in the handover phase, respectively.
$K_{sn}$, $R_{n}$ and $R_{s1}$, $R_{s2}$	Keys for SN and random number used in authentication process
MACsn, MACcs	MAC addresses of SN and CS, respectively
$T_{h}$, $R_{h}$	Timestamp and random number used in handover phase, respectively
H(.), ⊕, ‖	Cryptographic hash-function, bit-wise XOR, and concatenation, respectively

**Table 2:** Ban Logic notations.

Feature	Description
S∣≡X	*S* believes if the formula *X* is true
S∣∼X	*S* once said *X*
S⊲X	*S* sees *X*
$S\overset{k}{↔}H$	*k* is a shared-secret between *S* and *H*
$S\overset{K}{\Leftrightarrow }H$	*K* is a secret parameter known only *S* and *H*
#(X)	*X* is fresh.
${\left\{X\right\}}_{k}$	*X* is encrypted with the secret key *k*
〈X〉Y	*X* is combine with secret *Y*
S⇒X	*S* has jurisdiction over *X*
$\frac{S}{H}$	If *S* is true then *H* is also true

**Table 3:** Ban Logic inference rules.

Notation			Description
Message-Meaning-Rule			$\frac{S|\equiv S\overset{k}{↔}H,S⊲{\left\{X\right\}}_{k}}{S|\equiv H|∼X}$
Jurisdiction-Rule			$\frac{S|\equiv H\to X,S|\equiv H|\equiv X}{S|\equiv X}$
Belief-Rule			$\frac{S|\equiv (X,Y)}{S|\equiv X}$
Nonce-Verification-Rule			$\frac{S|\equiv \#(X),S|\equiv H|∼X}{S|\equiv H|\equiv X}$
Freshness-Rule			$\frac{S|\equiv \#(X)}{S|\equiv \#(X,Y)}$

**Table 4:** Simulation parameters.

Parameter					Size (Bits)
Encryption Algorithm					ASCON-128a
$ID_{sn}$					64
$SP_{}$					64
$ID_{cs}$					64
$SID_{sn}$					64
$SID_{ldr}$					64
timestamp					32
HASH Function					SHA-256
Random numbers					64

**Table 5:** Comparison of security properties.

	SAKES	EAKES6Lo	S6AE
Header Verification	×	×	✓
Replay attack	✓	✓	✓
Compromised attack	×	✓	✓
IP-Spoofing attack	×	×	✓
Unlinkability	×	✓	✓
Forward secrecy	×	✓	✓
Sybil attack	✓	✓	✓
Impersonation attack	✓	✓	✓
DOS attack	✓	✓	✓
MITM attack	✓	✓	✓
Identity Privacy Preservation	×	×	✓
Mutual authentication	✓	✓	✓
Mobility	×	✓	✓

**Table 6:** Computational overheads.

Scheme	SN	6LDR	6LAR	CS	Total Time
SAKES	$3T_{aes}+T_{sha}$	$2T_{exp}+2T_{aes}+T_{sha}$	-	$T_{exp}+3T_{aes}+2T_{sha}$	$3T_{exp}+8T_{aes}+4T_{sha}\approx 58.6044\;$ ms
EAKES6Lo	$2T_{aes}+T_{sha}+T_{sg}$	$T_{sv}$	$T_{sha}+T_{aes}$	$3T_{aes}+2T_{sha}+T_{sv}$	$5T_{aes}+4T_{sha}+2T_{sv}+T_{sg}\approx 17.2494\;$ ms
S6AE	$2T_{ascon}+5T_{sha}+7T_{xor}$	$T_{xor}$	$T_{sha}$	$2T_{ascon}+7T_{sha}+14T_{xor}$	$4T_{ascon}+13T_{sha}+22T_{xor}\approx 0.6643\;$ ms

**Table 7:** Communication overhead.

	Security Schemes		
Exchanged Messages	EAKES6Lo	SAKES	S6AE
SN→6LDR	672 bits	688 bits	496 bits
6LDR→SN	784 bits	2176 bits	528 bits

**Table 8:** Transmission energy consumption.

Proposed Scheme					Energy Consumption
S6AE					0.785 mJ
EAKES6Lo					1.11888 mJ
SAKES					2.25792 mJ

**Table 9:** Storage cost comparison.

Storage Cost	SAKES	EAKES6Lo	S6AE
Sensor (SN)	272 bytes	88 bytes	46 bytes
Server (CS)	272 bytes	80 bytes	54 bytes

**Table 10:** Handover overhead.

	Computational Overhead		Communication Overhead			
Scheme	Computational Time	Time Cost (ms)	SN→6LDR2	6LDR2→SN	No. of Messages	Energy Cost (mJ)
EAKES6Lo	$6T_{aes}+4T_{m}+T_{sg}+T_{sv}+2T_{sha}$	11.9366	704 bits	672 bits	6	1.05
S6AE	$2T_{ascon}+4T_{sha}$	0.2544	480 bits	418 bits	6	0.68
SAKES	n/a	-	n/a	n/a	n/a	n/a

## References

[1] Miguel M., Jamhour E., Pellenz M., Penna M. (2018). SDN architecture for 6LoWPAN wireless sensor networks. Sensors.

[2] Nait Hamoud O., Kenaza T., Challal Y. (2018). Security in device-to-device communications: A survey. IET Netw..

[3] Gomes T., Salgado F., Pinto S., Cabral J., Tavares A. (2017). A 6LoWPAN accelerator for Internet of Things endpoint devices. IEEE Internet Things J..

[4] Gomez C., Paradells J., Bormann C., Crowcroft J. (2017). From 6LoWPAN to 6Lo: Expanding the universe of IPv6-supported technologies for the Internet of Things. IEEE Commun. Mag..

[5] Hennebert C., Santos J.D. (2014). Security protocols and privacy issues into 6LoWPAN stack: A synthesis. IEEE Internet Things J..

[6] Li Y., Wang X. (2020). Green content communications in 6LoWPAN. IET Netw..

[7] Kushalnagar N., Montenegro G. (2007). Transmission of IPv6 packets over IEEE 802.15. 4 networks. IEEE Commun. Mag..

[8] Ishaq I., Carels D., Teklemariam G.K., Hoebeke J., Abeele F.V.D., Poorter E.D., Moerman I., Demeester P. (2013). IETF standardization in the field of the Internet of Things (IoT): A survey. J. Sens. Actuator Netw..

[9] Yeole A., Kalbande D., Sharma A. (2019). Security of 6LoWPAN IoT Networks in hospitals for medical data exchange. Procedia Comput. Sci..

[10] Sha K., Wei W., Yang T.A., Wang Z., Shi W. (2018). On security challenges and open issues in Internet of Things. Future Gener. Comput. Syst..

[11] Butun I., Österberg P., Song H. (2019). Security of the Internet of Things: Vulnerabilities, attacks and countermeasures. IEEE Commun. Surv. Tutor..

[12] Raza S., Duquennoy S., Chung T., Yazar D., Voigt T., Roedig U. Securing communication in 6LoWPAN with compressed IPsec. Proceedings of the 2011 International Conference on Distributed Computing in Sensor Systems and Workshops (DCOSS).

[13] Raza S., Seitz L., Sitenkov D., Selander G. (2016). S3K: Scalable security with symmetric keys-DTLS key establishment for the Internet of Things. IEEE Trans. Autom. Sci. Eng..

[14] Chuang M.C., Lee J.F., Chen M.C. (2012). SPAM: A secure password authentication mechanism for seamless handover in proxy mobile IPv6 networks. IEEE Syst. J..

[15] Perrig A., Szewczyk R., Tygar J.D., Wen V., Culler D.E. (2002). SPINS: Security protocols for sensor networks. Wirel. Netw..

[16] Hussen H.R., Tizazu G.A., Ting M., Lee T., Choi Y., Kim K.H. SAKES: Secure authentication and key establishment scheme for M2M communication in the IP-based wireless sensor network (6LoWPAN). Proceedings of the 2013 Fifth International Conference on Ubiquitous and Future Networks (ICUFN).

[17] Qiu Y., Ma M. (2016). A mutual authentication and key establishment scheme for M2M communication in 6LoWPAN networks. IEEE Trans. Ind. Inform..

[18] Roselin A.G., Nanda P., Nepal S. Lightweight authentication protocol (LAUP) for 6LoWPAN Wireless Sensor Networks. Proceedings of the 2017 IEEE Trustcom/BigDataSE/ICESS.

[19] Wang X., Mu Y. (2017). Communication security and privacy support in 6LoWPAN. J. Inf. Secur. Appl..

[20] Glissa G., Meddeb A. (2019). 6LowPSec: An end-to-end security protocol for 6LoWPAN. Ad Hoc Netw..

[21] Hui J., Thubert P. Compression Format for IPv6 Datagrams over IEEE 802.15.4-Based Networks. https://www.hjp.at/doc/rfc/rfc6282.html.

[22] Dolev D., Yao A. (1983). On the security of public key protocols. Trans. Inform. Theory.

[23] Dobraunig C., Eichlseder M., Mendel F., Schläffer M. ASCON v1. 2. https://competitions.cr.yp.to/round3/asconv12.pdf.

[24] Fivez M. (2016). Energy Efficient Hardware Implementations of CAESAR Submissions. Master’s Thesis.

[25] Diehl W., Abdulgadir A., Farahmand F., Kaps J.P., Gaj K. (2018). Comparison of cost of protection against differential power analysis of selected authenticated ciphers. Cryptography.

[26] Adomnicai A., Fournier J.J., Masson L. (2018). Masking the lightweight authenticated ciphers ACORN and ASCON in software. IACR.

[27] Pundir S., Wazid M., Singh D.P., Das A.K., Rodrigues J.J.P.C., Park Y. (2020). Intrusion Detection Protocols in Wireless Sensor Networks Integrated to Internet of Things Deployment: Survey and Future Challenges. IEEE Access.

[28] Yang Y., Wu L., Yin G., Li L., Zhao H. (2017). A Survey on Security and Privacy Issues in Internet-of-Things. IEEE Internet Things J..

[29] Khan R., Kumar P., Jayakody D.N.K., Liyanage M. (2019). A survey on security and privacy of 5G technologies: Potential solutions, recent advancements and future directions. IEEE Commun. Surv. Tutor..

[30] Burrows M., Abadi M., Needham R.M. (1989). A logic of authentication. R. Soc. Open Sci..

[31] Armando A., Basin D., Boichut Y., Chevalier Y., Compagna L., Cuéllar J., Drielsma P.H., Héam P.C., Kouchnarenko O., Mantovani J. (2005). The AVISPA Tool for the Automated Validation of Internet Security Protocols and Applications.

[32] Automated Validation of Internet Security Protocols and Applications AVISPA. http://www.avispa-project.org/.

[33] Kivinen T., Kojo M. More Modular Exponential (MODP) Diffie-Hellman Groups for Internet Key Exchange. https://www.hjp.at/doc/rfc/rfc3526.html.

[34] De Meulenaer G., Gosset F., Standaert F.X., Pereira O. On the energy cost of communication and cryptography in wireless sensor networks. Proceedings of the 2008 IEEE International Conference on Wireless and Mobile Computing, Networking and Communications.

